# Digital IAPT: the effectiveness & cost-effectiveness of internet-delivered interventions for depression and anxiety disorders in the Improving Access to Psychological Therapies programme: study protocol for a randomised control trial

**DOI:** 10.1186/s12888-018-1639-5

**Published:** 2018-03-02

**Authors:** Derek Richards, Daniel Duffy, Brid Blackburn, Caroline Earley, Angel Enrique, Jorge Palacios, Matthew Franklin, Gabriella Clarke, Sarah Sollesse, Sarah Connell, Ladislav Timulak

**Affiliations:** 1 Cinical Research & Innovation, SilverCloud Health, Dublin, Ireland; 2 E-mental Health Research Group, School of Psychology, University of Dublin, Trinity College, Dublin, Ireland; 3 HEDS, ScHARR, The University of Sheffield, Sheffield, S1 4DA UK; 4 Berkshire NHS Foundation Trust, London, Berkshire UK

**Keywords:** Depression, Anxiety, IAPT, iCBT, RCT, Internet-delivered

## Abstract

**Background:**

Depression and anxiety are common mental health disorders worldwide. The UK’s Improving Access to Psychological Therapies (IAPT) programme is part of the National Health Service (NHS) designed to provide a stepped care approach to treating people with anxiety and depressive disorders. Cognitive Behavioural Therapy (CBT) is widely used, with computerised and internet-delivered cognitive behavioural therapy (cCBT and iCBT, respectively) being a suitable IAPT approved treatment alternative for step 2, low- intensity treatment. iCBT has accumulated a large empirical base for treating depression and anxiety disorders. However, the cost-effectiveness and impact of these interventions in the longer-term is not routinely assessed by IAPT services. The current study aims to evaluate the clinical and cost-effectiveness of internet-delivered interventions for symptoms of depression and anxiety disorders in IAPT.

**Methods:**

The study is a parallel-groups, randomised controlled trial examining the effectiveness and cost-effectiveness of iCBT interventions for depression and anxiety disorders, against a waitlist control group. The iCBT treatments are of 8 weeks duration and will be supported by regular post-session feedback by Psychological Wellbeing Practitioners. Assessments will be conducted at baseline, during, and at the end of the 8-week treatment and at 3, 6, 9, and 12-month follow-up. A diagnostic interview will be employed at baseline and 3-month follow-up. Participants in the waitlist control group will complete measures at baseline and week 8, at which point they will receive access to the treatment. All adult users of the Berkshire NHS Trust IAPT Talking Therapies Step 2 services will be approached to participate and measured against set eligibility criteria. Primary outcome measures will assess anxiety and depressive symptoms using the GAD-7 and PHQ-9, respectively. Secondary outcome measures will allow for the evaluation of long-term outcomes, mediators and moderators of outcome, and cost-effectiveness of treatment. Analysis will be conducted on a per protocol and intention-to-treat basis.

**Discussion:**

This study seeks to evaluate the immediate and longer-term impact, as well as the cost effectiveness of internet-delivered interventions for depression and anxiety. This study will contribute to the already established literature on internet-delivered interventions worldwide. The study has the potential to show how iCBT can enhance service provision, and the findings will likely be generalisable to other health services.

**Trial registration:**

Current Controlled Trials ISRCTN ISRCTN91967124. DOI: 10.1186/ISRCTN91967124. Web: http://www.isrctn.com/ISRCTN91967124.

Clinicaltrials.gov: NCT03188575. Trial registration date: June 8, 2017 (prospectively registered).

## Background

According to the Global Burden of Disease (GBD) study, depression and anxiety disorders contribute the highest degree of disability amongst all mental and substance abuse disorders [1]. Both anxiety and depressive disorders contribute to increased societal costs through higher health care utilisation [2, 3], as well as absenteeism from work [4]. In Europe, the total cost of mood disorders in 2010 was €113.4 billion, comprising direct health care costs and indirect costs including unemployment, sickness benefit, lost productivity, and early retirement [5]. In England, approximately 1 in 6 adults have a common mental disorder [6], and mental health disorders / difficulties have been estimated to represent 23% of the total cause of disability, higher than other conditions such as cancer and CHD [7].

### Treatment of depression and anxiety

Psychological therapies have been demonstrated to be equally as effective as anti-depressant medication [8] and a recent meta-analysis of 34 studies shows a consistent patient preference for psychological vs. pharmacological treatment [9]. Furthermore, psychological therapies such as Cognitive Behavioural Therapy (CBT) have been shown to be clinically and cost effective [10, 11] and has demonstrated effectiveness in treating, maintaining progress, and preventing relapse in both depression and anxiety disorders [12–15].

Psychiatric disorders are seldom the principal reason for GP visits, and yet in one primary care study, depression was encountered in upwards of 31% of consultations, with anxiety detected in approximately 19% of consultations [16]. However, once identified, a multitude of factors prevent individuals from accessing evidence-based psychological interventions, including long waiting lists and a shortage of trained professionals [17]. A number of studies have investigated how depression and anxiety disorder treatment in primary and specialised care settings can be improved [18] by means of ‘collaborative care’ [19], ‘disease management’ approaches, and ‘stepped care’ initiatives [20]. Within stepped care, professionally guided low-intensity self-help interventions have become an attractive evidence-based alternative. Low-intensity interventions refer to those that require less time with a specialist therapist and include bibliotherapy, guided self-help, and internet-delivered CBT interventions [21].

### Improving access to psychological therapies

The Improving Access to Psychological Therapies (IAPT) programme involves a five-step approach to psychological care for people with depression and anxiety within the National Health Service (NHS) mental health services [22]. Stepped care models match treatment intensity to client needs by providing the least intrusive and most effective intervention for the client upon entering services. This allows for the effective management of resources, thus increasing access [23].

Step 2 of IAPT allows access to evidence-based treatments by delivering low-intensity psychological support to patients presenting with mild to moderate symptoms of depression and anxiety. These interventions are often delivered by Psychological Wellbeing Practitioners (PWPs), who are predominantly graduate psychologists with further training in delivering low-intensity CBT-based interventions [22].

A key aspect of the success of the IAPT model is its ability to report on outcomes. All services have a requirement to collect data on patients, access times, waiting times, dropout, treatment type, clinical outcomes, employment status, and work and social adjustment. Together, these outcomes collectively inform the success of the IAPT service.

As recommended by the National Institute for Health and Care Excellence [10, 11], the IAPT programme additionally offers computerised cognitive behavioural therapy (cCBT) as a low-intensity, Step 2 intervention for individuals presenting with mild to moderate symptoms of depression and/or anxiety. cCBT and internet-delivered cognitive behaviour therapy (iCBT) are delivered by PWPs, whose support is offered by means of electronic or telephone communication. Digital services in IAPT have been implemented at step 2 to help overcome the common barriers to accessing mental health treatment, such as costs and long waiting lists. There is certainly room for significant growth in these much-needed digital applications in IAPT, as digital use for treatment purposes is at less than 2% [24].

There is now a substantial body of research that supports the efficacy of online delivered CBT for depression and anxiety disorders [25–27] and findings have been transferred into clinical practice. This presents an opportunity to examine the effectiveness of iCBT in a natural setting.

### Cost effectiveness of iCBT

Early cost-effectiveness studies suggested that there could be large public health savings with the use of cCBT for treating depression and anxiety in mental health services [28]. In fact, the National Institute for Health and Care Excellence recommended the use of computerised CBT interventions partially based on this data [10, 11].

A recent systematic review of economic evaluations of internet-delivered interventions for mental health highlighted that interventions for depression and anxiety demonstrated cost-effectiveness compared to unguided interventions, waiting list controls, treatment as usual, group CBT, and telephone counselling [29]. The authors also make a call for future studies to firmly establish the cost-effectiveness of internet-delivered interventions.

### Mechanisms of change in iCBT

Understanding the factors that account for therapeutic changes is a key factor in providing the best patient care and assists in the pathway toward improving psychological treatments [30]. Despite the large body of research on mechanisms of change in traditional CBT, little has been achieved to date in understanding the mechanisms that drive change and their relative contribution to outcomes in iCBT [31]. Mechanisms of change comprise both general and specific factors: general factors include elements such as the therapeutic alliance, credibility of treatment, and belief about the potential of the intervention; specific factors include cognitive or meta-cognitive mechanisms, such as change in dysfunctional attitudes or repetitive negative thinking styles, improvement in emotion regulation abilities, cognitive and behavioural treatment skills usage, and therapist behaviours [32].

#### Aims

The overarching aim of the present study is to implement and evaluate the long-term effectiveness & cost-effectiveness of internet-delivered interventions for depression and anxiety disorders within IAPT services. The specific research questions include the following:

- Are the SilverCloud *Space from Depression and Space from Anxiety* internet-delivered interventions in IAPT effective in treating depression and anxiety disorders, and maintaining outcomes at follow-up, compared to waiting-list groups?
- Are the SilverCloud *Space from Depression and Space from Anxiety* internet-delivered interventions cost-effective in treating depression and anxiety disorders in IAPT?
- Are mediators/mechanisms of change and maintenance associated with both post-treatment and long-term effectiveness of internet-delivered interventions for depression and anxiety?

We hypothesize that: a) the iCBT interventions will be more effective than the waiting-list group in treating depression and anxiety disorders; b) the iCBT interventions will be cost-effective compared to the waiting-list group in treating depression and anxiety; c) we expect that mediators/ mechanisms of change including, repetitive negative thinking styles, emotion regulation skills, therapeutic alliance, credibility and expectancy in iCBT, CBT skills usage and therapist behaviours will be identified as positively impacting post-treatment outcomes and maintenance of gains in iCBT.

## Methods

### Study setting

#### Study setting

This naturalistic study will be conducted within Berkshire Healthcare NHS Foundation Trust through Talking Therapies, an NHS IAPT provider, that serves a population of 900,000 across 7 Clinical Commissioning Groups (CCGs), all of which are demographically and economically diverse, ranging from rural West Berkshire to urban commuter towns close to London. Talking Therapies aims to provide an easily accessible and clinically effective service for those within the community who suffer from anxiety and depressive disorders. These objectives were met in the last year by providing a stepped care model of mental health services to over 11,000 individuals. Those wishing to access the service can do so through self-referral, GP referral, or referral from allied services. If suitable, clients are offered treatment either at Step 2 or Step 3 based on their need. Step 2 services include low-intensity CBT-based treatments such as guided self-help, iCBT and group treatment, supported by trained psychological wellbeing practitioners (PWP). Step 3 services include high-intensity face-to-face CBT and counselling interventions, delivered by a variety of counsellors, CBT therapists and psychologists.

#### Trial design

A parallel-groups, randomised controlled trial design will be used to examine the effectiveness and cost-effectiveness of internet-delivered interventions for depression and anxiety disorders against a waiting list control group. Based on the primary diagnosis obtained from the Mini International Neuropsychiatric Interview 7.0.2 (M.I.N.I), participants will be randomised into one of two groups: one for depression and another for anxiety.

**Fig. 1:**
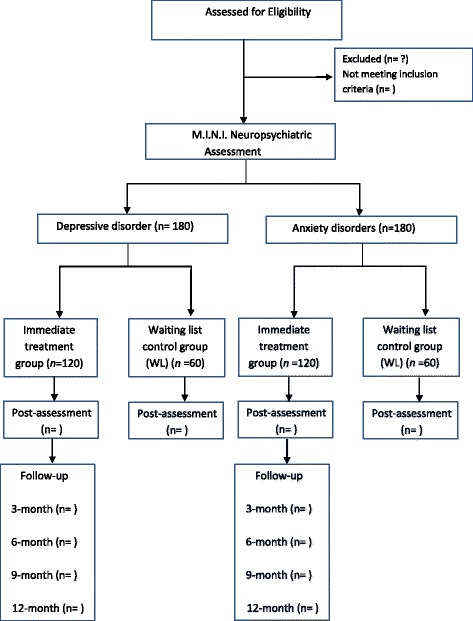
Participant flow – CONSORT

Randomisation will follow a 2:1 ratio. For ethical reasons, participants in the control group will receive access to the internet intervention after the waiting list period (8 weeks). The study will be conducted following the CONSORT statement [33], the CONSORT extension for web-based interventions (CONSORT-EHEALTH) [34] and the SPIRIT guidelines (Standard Protocol Items: Recommendations for Interventional Trials) [35]. The SPIRIT checklist can be found in Additional file 1. The study protocol, information on the study, informed consent, and trial related documents have been submitted and approved by the NHS England Research Ethics Committee [REC Reference: 17/NW/0311]. The study flowchart is shown in Fig. 1.

#### Sample size

Sample size was determined separately for primary presentations of anxiety disorders and depression for the trial. Using the software program G-power [36] and based on a moderate between group effect size of *d* = 0.5 with a power of 80%, an alpha error of 0.05, and a 2:1 randomisation procedure into immediate treatment and a corresponding waiting list control group. This calculation returned a total sample of 144 participants in the depression arm (96 in the treatment group and 48 in the WL group) and the same for anxiety, leading to a total of 288 participants. To ameliorate against attrition, a 25% uplift was added, resulting a total sample size of 360. Therefore, this number translates to a ratio of *n* = 120 in the treatment group and a corresponding *n* = 60 in the control group for depression, and the same ratio for anxiety. The 2:1 randomisation procedure was implemented to reduce the likelihood of having many people waiting for treatment after presenting to the IAPT service.

#### Eligibility criteria

##### Patient eligibility

All adult users of the Berkshire NHS Trust IAPT Talking Therapies Step 2 services will be eligible to participate. Upon screening participants, the inclusion and exclusion criteria illustrated in Table 1 will be applied. Suitability for an internet intervention is assessed by the PWP based on the willingness of the participants to engage on the iCBT intervention, presence of low to moderate levels of anxiety and depression, no suicidal or self-harm risk, and having internet access.

**Table 1:** Inclusion and exclusion criteria

Inclusion Criteria	Exclusion Criteria
Minimum age of 18 years	Suicidal intent/ideation: score > 2 on PHQ-9 question 9
A score of ≥9 on PHQ-9 and/or a score of ≥8 on GAD-7	Psychotic illness
Suitable for an internet-delivered intervention (iCBT)	Currently in psychological treatment for depression and/or anxiety
	Alcohol or drug misuse
	Previous diagnosis of an organic mental health disorder

*Note: PHQ-9* Patient Health Questionnaire-9, *GAD-7* Generalised Anxiety Disorder-7, *iCBT* internet-delivered cognitive behaviour therapy

#### Clinician eligibility

All Psychological Wellbeing Practitioners (PWP), who give clinical support to trial participants, will be eligible to participate in the research that will explore the therapeutic alliance online and the use of therapist behaviours and their relationship to outcomes. PWPs will have received training in the use of the iCBT intervention, the content of the programmes, as well as in delivering feedback when inducted into the service. In addition, all Case Managers providing supervision to clinical supporters of trial participants will be invited to take part in the research and complete the fidelity checklist, which assesses the congruence between the PWP’s written reviews of the clients and the module content they are completing.

##### Recruitment

ᅟ

#### Client recruitment

Recruitment will begin in June 2017 and will continue for 9 months until the numbers are reached or exceeded. Given that prevalence of anxiety disorders is higher than depression, it might be the case that the numbers on the anxiety arm will be reached before the depression arm. If necessary, recruitment on the anxiety arm will be stopped, but patients with primary diagnosis of a specific anxiety disorder will be still offered treatment.

Regarding the recruitment process, firstly, individuals will be given an initial assessment by phone with a Psychological Wellbeing Practitioner (PWP) clinician at Berkshire IAPT service. The assessment will determine if an individual meets the eligibility criteria, and an initial indication of depression, anxiety, or comorbid depression and anxiety will be advised by the PWP. The PWP will then describe the trial and invite the client to participate. Those interested will be assigned a further telephone appointment, to complete a structured clinical interview, the Mini-International Neuropsychiatric Interview (M.I.N.I.) [37]. They will also receive an email with information detailing the study and a link to give consent by means of a digital signature. Upon giving consent, participants will complete the primary and secondary outcomes for the study online (described below). During the M.I.N.I. appointment, the PWP will reiterate details of the research and the programme, describing what is involved and the importance of their participation in the trial. Participants will be allocated to the depression or anxiety arm based on the outcome of the M.I.N.I., and then randomized to the treatment or the waiting-list group. They will be informed of the outcome of randomisation during this appointment. Participants will then receive an email confirming whether they are in the immediate treatment group or waiting list group, and how to proceed. All participants will be informed they are free to withdraw if they no longer wish to take part in the trial. In this case, they will be removed from the trial and they will be offered treatment as usual.

#### Risk management

At assessment, clients are assessed for risk in line with routine clinical practice. This includes an assessment of whether patients can maintain their safety whilst on the waiting list. Those who exceed a clinically determined cut-off for “low-risk” in terms of suicide and/or self-harm on the screening questions will not be eligible to participate in the study and will be referred for additional support (i.e. thoughts of suicide/self-harm may be present but with no active plans, a low level of intentions to act on these thoughts with identified protective factors). Integrated risk measures in the SilverCloud programme allow for monitoring by supporters of any changes in risk for patients throughout the programme. For example, if the patient scores above > 2 on the suicide and self-harm item of the PHQ-9, the user will be prompted to complete 3 qualifying questions in relation to plans, intentions, preparations and protective factors against suicide and self-harm. If required, an alert will be sent through to their clinician, which can then be escalated appropriately within the established clinical governance structure. It is important to note that SilverCloud will not be presented to patients as a programme capable of providing crisis support, and this will be further emphasised through informed consent and the user contract. Once patients have finished the supported period of the intervention, their outcomes on the PHQ-9 and GAD-7 will determine whether they will be appropriately discharged or referred on for further treatment within Berkshire NHS Trust IAPT service.

#### Clinician recruitment

All PWP clinicians will be invited to a brief information session where the trial and the objectives will be fully described. They will be invited to participate as clinical supporters for the iCBT (*Space from Depression* and *Space from Anxiety*) interventions, but also as research participants themselves. As research participants PWPs will:

1. Electronically sign informed consent
2. Complete STAR-C and therapist behaviours checklist after each review session with a client
3. Participate in a semi-structured interview post-trial about their experience of the therapeutic alliance and their use of therapist behaviours.

#### Randomisation and blinding

Based on the outcome from the M.I.N.I., PWP’s will establish a primary diagnosis of depression or anxiety disorder and allocate participants to the depression or anxiety arm, respectively. Participants will be randomised using an algorithm performed by a computer scientist and executed independently of the research team, employing random permuted blocks using block sizes of 9, and including stratification within a 2:1 allocation ratio between the treatment group and the waiting list control group. The PWP who carries out the support and assessment of the patients cannot be blinded to allocation for practical reasons.

Intervention: SilverCloud platform.

SilverCloud Health are global leaders in the development of computerised psychological interventions for depression, anxiety, stress and comorbid long-term conditions. Both treatments, depression and anxiety, are delivered on a Web 2.0 platform using media-rich interactive content. This platform employs several innovative and engaging strategies for improving the user experience. The programme content is delivered in a non-linear fashion.

#### Space from depression

The online intervention ‘*Space from Depression*’ is a seven-module online CBT-based intervention for depression, delivered on a Web 2.0 platform using media-rich interactive content. Programme content is delivered in a non-linear fashion. Each module takes roughly 1 h to complete, and it is recommended one module be completed per week. The structure and content of the programme modules follow evidence-based CBT principles. The treatment is comprised of established cognitive and behavioural components including self-monitoring, self-control desensitisation, gradual stimulus control, thought recording, behavioural activation, cognitive restructuring, relaxation training and challenging core beliefs. In addition, given the frequent comorbidity of depression with anxiety symptoms, the platform provides PWPs with an option to deliver a mixed programme comprising the core depression modules delivered alongside a module about worry management. Each module is structured to incorporate introductory quizzes, videos, informational content, interactive activities, as well as homework suggestions and summaries. In addition, personal stories and accounts from other users are incorporated into the presentation of the material. The intervention follows NICE guidelines for the treatment of depression and the intervention has been tested and proved efficacious [10, 11, 38].

#### Space from anxiety

The online intervention ‘*Space from Anxiety’* is a seven-module online CBT-based intervention for anxiety, delivered on a Web 2.0 platform using media-rich interactive content. Programme content is delivered in a non-linear fashion. Each module takes roughly 1 h to complete, and it is recommended one module be completed per week. The structure and content of the program modules follow established evidence-based principles of CBT for the treatment of anxiety disorders. The treatment comprises cognitive, emotional, and behavioural components that include self-monitoring, relaxation training, cognitive restructuring, and worry outcome monitoring. The intervention also delivers customisations to modules based on the different anxiety disorder presentations, for example, in social anxiety the modules include attention control and the personal stories are of individuals experiencing social anxiety. The getting started module for the core anxiety programme introduces the user to the cycle of anxiety and the emotional, cognitive and behavioural aspects of anxiety. The goal of the intervention is to help people with anxiety as a primary disorder manage their thoughts, emotions and behaviours to help them alleviate their symptoms. The intervention follows NICE guidelines for the treatment of anxiety [10, 11].

#### Waiting list control group

Participants in the waiting list control group receive no treatment for the duration of the first 8-weeks. At week 8, waiting-list participants will complete the primary and secondary outcome measures and begin their treatment with support from a PWP.

#### Support during treatment

Each participant will be assigned a PWP clinician who will monitor participants’ progress throughout the trial. Once assigned to the active treatment condition the participant will receive a message from their PWP clinician at their first login. This message welcomes them to the program, highlights aspects of the program, and encourages them in use of the program. Over the course of the 8-week supported intervention, on 6 separate occasions the PWP will login and review participants progress, leaving feedback for them and responding to the work they have completed. The basic share level allows supporters to view users’ goals for the week, key messages and progress points. If users wish to share more with their supporter, they can share journal entries. Each supporter will provide post-session feedback of between 10 and 15 min per participant per session, over the eight-week intervention period.

Supporters will be supervised by case managers, who are responsible for monitoring fidelity of the PWP to the content of the specific iCBT intervention. At each supervision meeting they will complete a fidelity checklist. The checklist is an assessment of the congruence between the content of the PWP’s online reviews and the CBT module that the client is progressing through. This will ensure that patients receive the appropriate evidence-based intervention and that each client receives a similar standard of intervention, augmenting the reproducibility of the study. Furthermore, the trial steering group will meet weekly with PWP managers to audit the trial conduct, its processes and overall progress.

### Measures

#### M.I.N.I. International neuropsychiatric interview 7.0 (MINI) [37]

The current version is 7.0 and is based both on the Diagnostic and Statistical Manual of Mental Disorders (DSM-5) and the International Classification of Diseases (ICD-10) criteria. The interview has been well validated and the administration of the interview by telephone has been validated. The Interview schedule will include the following modules: Module A (Major Depressive Episode), Module D (Panic Disorder), Module F (Social Anxiety Disorder), and Module N (Generalized Anxiety Disorder), in order to establish current depression and anxiety and specific anxiety presentations. For research purposes randomisation to either *Space from Depression* or *Space from Anxiety* will be based on the outcome from the M.I.N.I. In the event where a clear diagnosis is not available the clinician will decide on the diagnosis based on the outcome from the M.I.N.I. and clinical knowledge from the initial assessment (scores on the PHQ-9, GAD-7).

##### Primary outcome measures

ᅟ

#### Patient health Questionnaire-9 (PHQ-9) [39, 40]

The PHQ-9 is a self-report measure of depression that has been widely used in research and is a regular screening measure utilised in primary care and hospital settings. The PHQ-9 items reflect the diagnostic criteria for depression outlined by the Diagnostic and Statistical Manual of Mental Disorders, Fourth Edition – Text Revision (DSM–IV–TR) [41]. Summary scores range from 0 to 27, where larger scores reflect a greater severity of depressive symptoms. The PHQ-9 has been found to discriminate well between depressed and non-depressed individuals using the cut-off total score ≥ 10, with good sensitivity (88.0%), specificity (88.0%) and reliability (89, 39).

#### Generalized anxiety Disorder-7 (GAD-7) [42]

GAD-7 comprises 7 items measuring symptoms and severity of anxiety based on the DSM-IV diagnostic criteria for GAD. The GAD-7 has good internal consistency (α = .92) and good convergent validity with other anxiety scales [42]. Higher scores indicate greater severity of symptoms. The GAD-7 has increasingly been used in large-scale studies as a generic measure of change in anxiety symptomatology, using a cut-off score of 8 [43].

##### Secondary outcome measures

ᅟ

#### Diagnosis-specific measures

Participants with a principal diagnosis of anxiety and of specific anxiety including social anxiety, GAD, panic disorder or health anxiety will also complete the associated anxiety disorder specific measure depending on their main diagnosis.

##### IAPT phobia scales

The IAPT Phobia scales have been designed with the aim of capturing patients who may score below the clinical cut-off on the PHQ-9 and GAD-7 but whose lives may be significantly impaired by the presence of social anxiety, agoraphobia and specific phobias [44].

##### Social Phobia Inventory (SPIN) [45]

Consisting of 17 self-rated items for social anxiety disorder, this test asks the user to reflect over the past week and report on their experiences as laid out by the inventory, which assesses the domains of social anxiety disorder (fear, avoidance and physiological arousal). Scores are then totaled to produce a value that is representative of symptom severity on a continuum from none to very severe. Internal reliability for the SPIN has been placed at α = .95, with α values for the subscales ranging from .79–.85. [45].

##### Short Health Anxiety Inventory (HAI) [46]

This scale measures levels of health anxiety, which is characterised by the misinterpretation of bodily sensations as a serious illness. The shortened version of the scale has been constructed such that it is sensitive to both normal and severe levels of health anxiety. A meta-analysis of the inventory has yielded α values between .74–.96 [47].

##### Panic Disorder Severity Scale-Self Report (PDSS-SR) [48]

This scale measures severity of panic disorder and is considered a reliable tool in treatment outcome studies. It consists of seven items rated on a 5-point scale that ranges from 0 to 4. The items assess panic frequency, distress during panic, panic-focused anticipatory anxiety, and phobic avoidance of situations, phobic avoidance of physical sensations, impairment in work functioning, and impairment in social functioning. The total sum of the individual scores ranges from 0 to 28, where higher scores indicate greater severity. A score of 9 or above is indicative of caseness. It has displayed good psychometric properties [49].

##### The Penn State Worry Questionnaire (PSWQ) [50]

This questionnaire consists of 16 items and is considered a valid clinical measure of the worry characteristic of GAD. Each item is measured on a 5-point Likert scale (1 – not at all typical of me to 5 – very typical of me) and a total score ranging between 0 and 80 is calculated by summing all items. Psychometric evaluations have revealed a high internal consistency (α = 0.86 to 0.95) and test-retest reliability over four weeks (*r* = 0.74 to 0.93) [51]. The measure has also been found to successfully differentiate between patients with GAD and those with other anxiety disorders [52].

#### Functional impairment

##### Work and Social Adjustment (WSAS) [53]

This is a simple, reliable and valid measure of impaired functioning. It is a 5-item self-report measure that provides an experiential impact of a disorder from the patient’s point of view. It looks at how the disorder impairs the patient’s ability to function day to day on five dimensions: work, social life, home life, private life and close relationships.

#### Cost effectiveness

##### EuroQoL Five Dimension Five Level Version (EQ-5D-5 L) [54]

This measure is used to assess a person’s perception of their general health state and obtain a measure of quality adjusted life years (QALYs). Outcomes can be benchmarked against UK population norms. It covers five dimensions: mobility, self-care, usual activity, pain/discomfort and anxiety/depression, which are rated by the person on five levels of severity: no problems, slight problems, moderate problems, severe problems and extreme problems/unable to function within that domain.

##### Recovering Quality of Life – 10 item version (ReQoL-10) [55]

This recently developed measure is used to assess a person’s perception of their generalised mental well-being which is associated with recovering quality of life. The measure focuses on seven key themes associated with recovering quality of life: well-being; autonomy, control, choice; self-perception; relationships and belonging; hope; activity; physical health. It is composed of 10 questions (with one additional question focused on physical health) rated on a 5-point scale which describes a persons thoughts, feeling and activities over the last week.

##### Client Service Receipt Inventory (CSRI) [56]

This collects individuals’ use of health and social care resources. It comprises questions about health care utilization including inpatient and outpatient hospital services, community-based day services, primary and community care contacts and employment status. This version of the CSRI is routinely administered at the study site.

#### Satisfaction with treatment measures

##### Patient Experience Questionnaire (PEQ) [44]

This instrument will be used to assess patient experience and satisfaction. The PEQ contains several quantitative questions and open-ended questions that are used to assess participant’s views and satisfaction with service provision.

#### Mechanisms of change and maintenance

##### Scale to Assess Therapeutic Relationship – Patient version (STAR-P) [57]

This scale will be employed to assess patients’ experiences of the therapeutic relationship online. It will be administered to patients throughout treatment following each progress review with the clinician.

##### Scale to Assess Therapeutic Relationship – Clinical version (STAR-C) [57]

This will be employed to assess clinicians’ experiences of the therapeutic relationship online. The measure will be administered each time the clinician writes a review for their clients.

##### Positive Beliefs about Rumination Scale – Adapted Version (PBRS-A) [58]

This scale has 9 items that measure positive beliefs about repetitive negative thinking. Participants complete each item using a 4-point scale, 1 = *do not agree* to 4 = *agree very much*. The measure has good internal reliability (α = .89).

##### Emotion Regulation Questionnaire (ERQ) [59]

The ERQ is a 10-item scale that assesses individual differences in the habitual use of two emotion regulation strategies: cognitive reappraisal and expressive suppression. Each item is rated on a 7-point scale so that higher scores indicate higher use of reappraisal/ suppression. This scale has shown appropriate levels of internal reliability on both subscales, reappraisal and suppression (0.79 and 0.73, respectively).

##### Frequency of Actions and Thoughts Scale (FATS) [60]

The FATS is a 12-item scale, designed to assess the frequency with which CBT-related skills have been used during the previous week. Each item is measured on a 5-point scale (0 = not at all, 1 = one or two days; 2 = half the days; 3 = almost every day; 4 = every day). The FATS comprises four subscales, indicative of how much the person engaged in 1) cognitive restructuring, 2) social interaction, 3) rewarding behaviours and 4) activity scheduling in the past week; but also gives an overall score, indicative of overall CBT-related skills usages. Psychometric evaluations suggest high internal consistency of the total FATS scale (α = 0.86) and acceptable to high internal consistency of its subscales (α = 0.74 to 0.83). The FATS has been shown to be sensitive to change during iCBT and higher scores on the FATS have been linked to better treatment outcomes.

##### Psychological wellbeing practitioner behaviours checklist

A checklist of behaviours based on existing literature has been developed to assess the behaviours used by PWPs in their communication with patients. The checklist is based on known therapist behaviours and the items have been evaluated by PWPs for relevance. It will be an optional function within the SilverCloud platform that PWPs will be invited to complete each time they communicate with their patient.

##### Expectancy/credibility questionnaire

This is a one question item that assesses the clients level of credibility with the intervention and is based on the work in this area [61].

#### Dropout measure

Finally, a *dropout questionnaire* will assess participants’ reasons for dropout from treatment. It includes only one open question asking for details about the specific reasons for dropout.

The study measures and assessment times are summarised in Table 2.

**Table 2:** Study measures and assessment times

Measure	Assessment	Time of assessment
M.I.N.I. 7.0.2 for DSM-5	Diagnosis	BL and 3-FU
PHQ-9	Depression	BL, contin, Post-T, FU
GAD-7	Anxiety	BL, contin, Post-T, FU
IAPT Phobia Scale	Specific Phobia	BL, contin, Post-T, FU
WSAS	Functioning	BL, contin, Post-T, FU
SPIN	Social Phobia	BL, Post-T, FU
HAI	Health anxiety	BL, Post-T, FU
PSWQ	GAD	BL, Post-T, FU
PDSS	Panic	BL, Post-T, FU
EQ-5D-5 L	Health status	BL, Post-T, 6-FU, 9-FU, 12-FU
ReQol-10	Recovering quality of life/ mental wellbeing	BL, Post-T, 6-FU, 9-FU, 12-FU
CSRI	Resource utilisation	BL, Post-T, 6-FU
PEQ	Patient experience	Post-T
STAR-C	Therapeutic alliance	Throughout treatment, at each review of client
STAR-P	Therapeutic alliance	Throughout treatment, after each progress review with clinician
PBRS-A	Repetitive Negative Thinking	BL, week 4, Post-T, 3-FU
ERQ	Emotion Regulation	BL, Post-T
FATS	CBT-related skills usage	FU
Therapist behaviours checklist	Therapist behaviours	During treatment at review of client
Expectancy / credibility	Expectancy and credibility with the intervention	BL, week 4, Post-T
Dropout questionnaire	Reasons for dropout	3-FU

*BL* Baseline, *Post-T* Post-Treatment, *FU* All follow-ups, *3-FU* 3-month follow-up, *6-FU* 6-month follow-up, *contin* continuous, *PHQ-9* Patient health questionnaire-9, *GAD-7* Generalized anxiety disorder-7, *WSAS* Work and social adjustment, *SPIN* Social Phobia Inventory;` *HAI* Short health anxiety inventory, *PDSS* Panic disorder severity scale-self report, *PSWQ* Penn state worry questionnaire, *EQ-5D-5 l* EuroQol-5 Dimension-5 level, ReQoL-10 Recovering quality of life – 10 item version, *CSRI* Client services receipt inventory, *PEQ* Patient Experience questionnaire, *PBSR-A* Positive Beliefs about Rumination Scale – Adapted Version, *FATS* Frequency of Actions and Thoughts Scale, *ERQ* Emotion Regulation Questionnaire

##### Engagement and usage measures

The online system will collect anonymized descriptive data relating to engagement and usage of the service users with the platform. Data collected will include the number of modules completed, time spent in the platform, number of activities completed, number of minutes per log-in, number of sessions and length of sessions. A session is defined as an instance where a user logs on to the system. Session time will be always an imperfect calculation, as users may take breaks within a session, without formally log out of the system. To prevent this overestimation, periods of more than 30 min without interaction will be taken as one minute and periods of inactivity longer than 3 h will start the count on a new session. Use of different program components will be measured.

### Statistical analysis

The effectiveness of the internet-delivered interventions compared to control will be analysed using the intention to treat (ITT) approach. Participation levels will be monitored throughout the study and reasons for withdrawal or non-compliance of study subjects will be recorded to document bias associated with non-random protocol deviations. Any potential imbalances will be reported and considered fully in interpretation of the trial results. Missing data will be evaluated for randomness and imputed using multiple imputation procedures.

Differences in baseline primary and secondary outcome measures between treatment groups and waiting list control groups will be identified using χ^2^ tests for categorical variables and ANOVAs or t-tests for continuous variables. Efficacy of treatments over time will be measured using mixed effects models. To complement the post-hoc comparisons, the magnitude of change within and between the groups on the primary and secondary outcomes measures will be established using Cohen’s *d* statistic. Bonferroni corrected *p*-values will be reported for multiple comparisons. All statistical analyses will be conducted in SPSS, SAS and R. Results will be presented following CONSORT guidelines for reporting parallel group randomised trials.

The established IAPT definitions of recovery will be used in our analysis, where ‘recovery’ is determined by patients moving from ‘caseness’ at the beginning of the intervention (i.e. scoring ≥9 on the PHQ-9 or ≥8 on the GAD-7) to ‘non-caseness’ at the end of treatment (thus scoring below the cut-off). Two further established measures will be considered: *reliable improvement*, where the patient has a significant decrease in their PHQ-9 / GAD-7 score upon completion of their course of treatment; and *reliable recovery*, where the patient meets the criteria for both recovery and reliable improvement. Thus, a patient who moves from caseness to non-caseness, and does so by showing a significant decrease in their symptom scores, is said to have a reliable recovery [44].

Exploratory analyses, including the use of correlation and multiple regression, will be conducted to explore the role that mechanisms of change and maintenance measures play in the benefits obtained from the interventions. Moderation and mediation analyses may include the use of macros for SPSS and/or bootstrapping for exploring the role that mechanisms of change play on the outcomes.

Resource-use and subsequent costs will be estimated over the 12-month time horizon for the intervention group and for the 8-week waiting list time period for the control group. Healthcare resource will be valued using unit costs derived from available data sources [62–66].

For the purpose of the economic evaluation, EQ-5D-5 L and ReQoL-10 preference-based tariff score values will be used to elicit the quality adjusted life year (QALY) using the area under the curve (AUC) method to account for the collection of this data at multiple time points between baseline and 12-month follow-up in both trial-arms. The proposed economic analysis will be conducted for those with anxiety and those with depression separately and as a whole patient group. All economic analysis will be performed using Stata version 14 (or an updated version). Due to the shorter time horizon of the control groups (8 weeks based on a waiting list approach) compared to the intervention group (12 months with outcome assessment at 8 weeks, 6 months, 9 months and 12 months), methodological work will be carried out to assess the feasibility and uncertainty around extrapolating outcomes from the 8-week time period in the control group to a longer time horizon (e.g. 6-months, 9-months and 12-months) using survival analysis methods [67]. Cost per QALY analysis will be carried out over multiple time horizons (e.g. 8-weeks, 6-months, 9-months and 12-months) to address the uncertainty around decision making from using observed data (up to 8 weeks) and extrapolated outcomes (beyond 8 weeks) for the economic evaluation. Incremental mean point estimates of the difference in cost and QALYs between trial groups will be used to determine the incremental cost effectiveness ratio (ICER). ICER is defined here as the ratio between incremental costs and incremental effectiveness and the following formula will be employed:

$$ ICER=\frac{\mathrm{Cost}\kern0.5em \mathrm{active}\kern0.5em \mathrm{intervention}\kern0.5em \mathrm{group}\hbox{-} \mathrm{Cost}\kern0.5em \mathrm{control}\kern0.5em \mathrm{intervention}\kern0.5em \mathrm{group}}{\mathrm{Efectiveness}\kern0.5em \mathrm{or}\kern0.5em \mathrm{Utility}\kern0.5em \mathrm{active}\kern0.5em \mathrm{intervention}\kern0.5em \mathrm{group}\kern0.5em \hbox{-} \mathrm{Efectivenes}\kern0.5em \mathrm{or}\kern0.5em \mathrm{Utility}\kern0.5em \mathrm{control}\kern0.5em \mathrm{intervention}\kern0.5em \mathrm{group}}. $$

Statistical bootstrapping will be used to plot the cost-effectiveness acceptability curves (CEACs) for the purpose of describing the probability of cost-effectiveness of the intervention compared to usual care at willingness-to-pay thresholds normally used by decision makers (i.e. £20,000 per QALY). One-way sensitivity analysis will be used to describe the uncertainty around these estimates. If the trial results suggest there is considerable uncertainty around the cost-effectiveness of the intervention, a simple economic decision model will be designed to inform an expected value of information (EVI) analysis. These EVI results can be used to suggest the upper bound cost of future research around this area of care.

## Discussion

Internet-delivered interventions have evolved in the past years. Today we have robust technology platforms that can configure and deliver a range of intervention modules to meet the needs of the patient. SilverCloud Health is a global provider of mental health and wellbeing solutions online, and Berkshire NHS trust has been using SilverCloud interventions which were developed alongside clinicians at IAPT, and are NICE approved and suitable for use at Step 2.

Undertaking the principal aim of this trial will allow for a robust test of the effectiveness of iCBT in treating depression and anxiety symptoms, and the maintenance and cost-effectiveness of these treatment effects in the long-term. Positive results of these main outcome measures will allow iCBT to consolidate itself as not only a valid treatment option, but as an essential component to the care management pathway offered throughout all IAPT services. This should support the expansion of SilverCloud programmes as the leader in the delivery of digital interventions at Step 2 of IAPT. However, the relevance of the results will also likely have implications for the implementation and success of digital interventions for depression and anxiety in health and mental health care systems worldwide.

Investigating potential mediators and moderators of change will contribute to our understanding of key processes in achieving improvement using online-treatments for anxiety and depression. Mediators and moderators of iCBT outcomes have been relatively understudied, but exploring the roles of constructs such as rumination, emotion regulation, therapeutic alliance and CBT skills usage will provide insight into clinical change. This will potentially inform the tailoring of interventions to best address the needs of the targeted population, ultimately leading to the development of more effective treatments.

The proposed economic analysis will add to the current empirical literature in regards to evidence of the cost-effectiveness of internet-based mental health interventions (i.e. iCBT) and will be an innovative analysis of the SilverCloud iCBT interventions. In a context of health care provision where resources are already stretched, cost-effective interventions can only support the delivery of effective mental health services.

In summary, iCBT interventions are fast becoming a viable option for patients with depression and anxiety symptomatology, and are in turn improving the quality of the services which provide them. They can be beneficial both in costs and time management, and as technology moves swiftly so too must research continually and rapidly inform on the benefits of providing iCBT. This large-scale trial aims precisely to drive this research forward, thus improving the management and quality of life of an ever-growing patient population.

## Trial status

Recruitment began on 27th June 2017.

## Abbreviations

CBT: Cognitive Behavioural Therapy (CBT)
CCBT: Computerised Cognitive Behavioural Therapy
CHD: Coronary Heart Disease
CONSORT: Consolidated Standards of Reporting Trials
CSRI: Client Service Receipt Inventory
DSM-5: Diagnostic and Statistical Manual of Mental Disorders
DSM-IV: Diagnostic and Statistical Manual of Mental Disorders, Fourth Edition
EQ-5D-5 L: EuroQoL 5-Dimension 5-Level
ERQ: Emotion Regulation Questionnaire
FATS: Frequency of Actions and Thoughts Scale
GAD: Generalized Anxiety Disorder
GAD-7: Generalised Anxiety Disorder – 7-item
GBD: Global Burden of Disease
GP: General Practitioner
HAI: Short Health Anxiety Inventory
IAPT: Improving Access to Psychological Therapies
ICBT: Internet-based Cognitive Behavioural Therapy
ICD-10: International Classification of Diseases – 10th Revision
ITT: Intention To Treat
M.I.N.I.: Mini-International Neuropsychiatric Interview
NHS: National Health Service
NICE: National Institute for Health and Care Excellence
PBRS-A: Positive Beliefs about Rumination Scale – Adapted
PDSS-SR: Panic Disorder Severity Scale-Self Report
PEQ: Patient Experience Questionnaire
PHQ-9: Patient Health Questionnaire – 9-item
PSWQ: Penn State Worry Questionnaire
PWP: Psychological Wellbeing Practitioner
QALY: Quality-adjusted Life Year
RCT: Randomized Controlled Trial
ReQoL-10: Recovering Quality of Life – 10-Item
SPIN: Social Phobia Inventory
SPIRIT: Sharing Patients’ Illness Representations to Increase Trust
STAR-C: Scale to Assess Therapeutic Relationship – Clinical version
STAR-P: Scale to Assess Therapeutic Relationship – Patient version
WSAS: Work and Social Adjustment
